# Explainable machine learning unveils the key role of cooperation ability in school bullying and its gender-differentiated impact on cooperative atmosphere

**DOI:** 10.3389/fpsyt.2026.1784847

**Published:** 2026-06-04

**Authors:** Yawen Lv, Ziwen Zhou, Jinguo Li, Fang Fang, Hongyu Wang

**Affiliations:** 1 Graduate School, Bengbu Medical University, Bengbu, China; 2 School of Humanities and Health, Bengbu Medical University, Bengbu, China

**Keywords:** adolescent, cooperation, explainable machine learning, school bullying, social-emotional competence

## Abstract

**Background:**

School bullying is a widespread and urgent public health issue that threatens adolescents’ social and emotional development. Understanding its impact on social-emotional skills is critical for designing effective interventions. This study examines the association between school bullying and social-emotional skills using data from the Organisation for Economic Co-operation and Development (OECD)’s 2019 Study on Social and Emotional Skills (SSES) conducted in Suzhou, China.

**Methods:**

We analyzed cross-sectional survey data from 7,088 Chinese adolescents (52.7% male, 47.3% female; aged 10–16 years). A LightGBM model was used to assess the relative importance of social-emotional dimensions in distinguishing bullying status. We selected Cooperation Atmosphere (a school-contextual measure reflecting students’ collaborative behaviors and attitudes) as the dependent variable and employed feature selection before developing five machine learning regression models. SHAP analysis interpreted key features, with gender-specific analysis conducted, revealing distinct predictive patterns between males and females.

**Results:**

50.8% of adolescents experienced bullying. Cooperation Ability showed the highest predictive importance among the social-emotional dimensions. XGBoost performed best in predicting Cooperation Atmosphere (Training: R²=0.5042, RMSE = 0.5219; Test: R²=0.4347, RMSE = 0.5684). SHAP identified school belonging, competitive climate, and peer support as top predictors. Gender-specific analysis revealed different predictive patterns across male and female students.

**Conclusions:**

This study suggests that school bullying is closely associated with poorer cooperation-related functioning and identifies key modifiable factors for targeted interventions. Findings provide evidence for educators and policymakers to develop gender-specific strategies for improving school climate and reducing bullying.

## 1 Introduction

Bullying in schools is a major global public health issue that endangers the physical and mental health of adolescents (1, 2). It is commonly defined as repeated, intentional aggression by individuals or groups toward those with less power, manifested through intimidation, humiliation, or exclusion. Victims often experience long-term psychological, emotional, and educational harm (3, 4). Studies indicate that approximately 32% of children and adolescents experienced school bullying at least once in the month preceding the survey (5). A study in China reported a higher prevalence, with up to 66% of youths having experienced bullying and 37% having engaged in bullying behaviors (6). Furthermore, adolescents involved in school bullying are more likely to suffer from maladaptive outcomes, including depression, emotional disorders, academic difficulties, and reduced subjective well-being (7–9).

Social-emotional competence includes essential abilities such as emotional regulation, cooperation ability, and responsibility, which help individuals adapt to complex social environments (10). It significantly promotes adolescent learning and development (11). The OECD’s assessment framework categorizes social-emotional competencies into five domains: task performance, emotional regulation, cooperation ability, open-mindedness, and social engagement, encompassing 15 specific skills (12). This framework provides a comprehensive basis for evaluating these competencies systematically. In recent years, cultivating social-emotional skills has gained increasing emphasis in global educational policy and reform (13, 14).

Existing studies show a significant negative correlation between social-emotional competence and school bullying (15, 16). Individuals with lower competence—especially in emotional regulation, stress resilience, trust, and social preference—are not only more vulnerable to bullying but also lack the emotional resources and coping mechanisms to respond effectively (17). Among these, cooperation ability is particularly crucial, playing a key role in adolescents’ moral development, peer relationships, academic achievement, and social adaptation (18, 19). Importantly, cooperation is closely embedded in trustful and positive peer interactions (20, 21). Because school bullying is characterized by repeated aggression, exclusion, and power imbalance, it may be particularly harmful to the development and expression of cooperative tendencies in adolescence (21).

The cooperation atmosphere refers to the shared psychological and environmental setting within student groups that encourages and sustains cooperative behavior. It serves as a critical contextual factor for developing cooperation ability. In other words, cooperation atmosphere can be understood as the school-contextual manifestation of cooperation-related functioning, rather than merely an individual characteristic. Based on SSES 2019 data, this study evaluates cooperation-related functioning through two dimensions: “Students Collaborate with Each Other” (behavioral) and “Students Value Cooperation” (attitudinal). This conceptualization makes cooperation atmosphere a meaningful outcome when examining how relational and environmental factors within school settings shape adolescents’ cooperative experiences. Therefore, after identifying Cooperation Ability as the social-emotional domain most closely related to bullying, it is theoretically meaningful to further examine Cooperation Atmosphere as its contextual expression in the school environment. This conceptualization emphasizes that individual experiences of bullying can influence the broader school context, shaping students’ collaborative behaviors and attitudes within their peer groups. A strong cooperation ability within a supportive cooperation atmosphere can significantly reduce bullying instances while offering essential socialization opportunities for students’ social-emotional development (22).

However, school bullying—characterized by aggression and exclusion—may undermine peer relationships and trust, thereby posing a serious threat to the development of cooperation ability (23). Although the detrimental effects of bullying are theoretically established, large-scale empirical studies have not yet thoroughly examined its specific relevance to cooperation-related functioning or identified key factors shaping the cooperation atmosphere.

From a theoretical perspective, bullying may affect cooperation-related functioning at both the individual and contextual levels (24). At the individual level, repeated victimization may undermine interpersonal trust and reduce willingness to engage in reciprocal peer interactions (21). At the contextual level, bullying may also undermine the social conditions that support cooperation by damaging students’ sense of belonging and perceived support within the school environment (25). Taken together, these findings suggest a multilevel pathway through which bullying may be linked to cooperation-related functioning. Therefore, examining both cooperation ability and cooperation atmosphere may provide a more comprehensive understanding of how bullying is associated with adolescents’ social-emotional development.

Based on the above theoretical and empirical background, three hypotheses were proposed. First, school bullying would be negatively associated with adolescents’ social-emotional competencies, with cooperation-related functioning showing the strongest relevance among the major social-emotional domains. Second, cooperation atmosphere would be associated with relational and school-contextual factors, especially school belongingness, peer support, teacher support, and competitive climate. Third, the relative importance of key factors associated with cooperation atmosphere would differ between male and female students.

Therefore, this study aims to examine the association between school bullying and social-emotional competencies and to explore critical factors associated with the cooperation atmosphere as a contextual expression of cooperation-related functioning. To achieve this, we employed a two-stage machine learning approach using 2019 OECD SSES data from China, specifically from Suzhou, the only city in China included in the OECD SSES dataset. This choice provides a unique opportunity to examine social-emotional skills in Chinese adolescents, enhancing the relevance and applicability of the findings for local educational policy and intervention strategies. In the first stage, a LightGBM binary classification model was used to examine the relative importance of different social-emotional competencies in distinguishing bullying status. In the second stage, cooperation atmosphere was used as the dependent variable in multiple regression models, from which the best-performing model was selected. We applied explainable machine learning (XML) techniques, specifically SHAP (Shapley Additive Explanations), to interpret each variable’s contribution and direction of influence, providing transparency for readers unfamiliar with these methods.

Gender-stratified analyzes were also conducted to identify key factors affecting the cooperation atmosphere across different groups. This study provides empirical evidence and suggests potential strategies for educators to develop tailored interventions aimed at reducing bullying and enhancing cooperation ability by identifying critical predictors of the cooperation atmosphere and their gender-specific characteristics.

## 2 Methods

### 2.1 Research subjects and data sources

This study utilized data from the SSES conducted by the OECD in 2019. Detailed information regarding the survey is available on the OECD official website (https://www.oecd.org/education/ceri/social-emotional-skills-study/, accessed April 15, 2026). The SSES was implemented across multiple cities worldwide and included four participant groups: students, parents, teachers, and school principals. The global dataset comprises 61,010 participants. This research focused specifically on adolescents from Suzhou, China, from which key variables were extracted. After excluding cases with missing values in critical variables, a total of 7,088 Chinese adolescents aged 10 to 16 years were retained for final analysis. The sample included both male and female students and covered a range of ages. Participant characteristics such as family learning resources, parental education, school safety perception, and peer relationships are reported to provide an overview of the study population for international readers.

### 2.2 Ethical approval

The analysis for this study is based on secondary, anonymized data from the OECD SSES 2019, a publicly available dataset. The original survey obtained informed consent from all participants and their parents or guardians in the case of minors. As this research did not involve direct interaction with human subjects or access to identifiable private information, it was exempt from ethical review in accordance with the policies of the Bengbu Medical University Research Ethics Committee.

### 2.3 Variable definition and measurement

This study consisted of two parts, each with distinct dependent and covariate variables. In the first part, which used binary modeling, the dependent variable was “school bullying.” It was measured using four questionnaire items: STQM03901 (Other students made fun of me), STQM03902 (I was threatened by other students), STQM03903 (Other students took away or damaged something belonging to me), and STQM03904 (I was hit or pushed by other students). Responses were scored from 1 to 4, corresponding to: “Never or almost never,” “A few times a year,” “A few times a month,” and “Once a week or more frequently.” The total score ranged from 4 to 16, with a score above 4 indicating exposure to school bullying. This variable was then coded as binary (yes/no). The covariate in this part was social-emotional competence, a multidimensional construct including five domains: open-mindedness, task performance, social engagement, Cooperation Ability, and emotion regulation. Each domain was represented by the sum of three specific social-emotional skills, with each skill measured by the total score of 8 items on a 5-point Likert scale. The social-emotional competence section included 120 items in total (see **Supplementary Table 1** for details).

In the second part, which involved regression modeling, the dependent variable was “Cooperation Atmosphere,” assessed through two items: STQM03801 (Students seem to value cooperation) and STQM03802 (Students seem to be cooperating with each other). Responses were scored on a scale from 1 to 4, corresponding to: “Never,” “Sometimes,” “Often,” and “Almost always.” This yielded a total score ranging from 2 to 8 for the two items, with higher scores indicating a more positive Cooperation Atmosphere. Covariates, sourced from the SSES 2019 database, were categorized into five groups: (1) personal behaviors and activities (e.g., time spent on exercise, social activities, and video games); (2) psychological and emotional states (e.g., life satisfaction, emotional well-being, and perceived school safety); (3) social relationships and support (e.g., peer friendliness, family intimacy, teacher support, and sense of school belonging); (4) family environment factors (e.g., parental expectation pressure and parental education level); and (5) other relevant variables (e.g., self-rated health and competitive atmosphere).

### 2.4 Data preprocessing

This study used data from 61,010 participants obtained from the OECD SSES 2019 database. We excluded samples with missing values in key variables, as well as variables that had a missing rate exceeding 20%, resulting in a final analytical sample of 7,088 individuals. Missing values in the remaining variables were imputed using the IterativeImputer from scikit-learn, which applies a multivariate imputation by chained equations (MICE) approach to preserve relationships among variables and reduce bias. Subsequently, the dataset was standardized using Z-scores to remove scale differences between features, improving comparability and reducing the risk of overfitting. All preprocessing steps were performed exclusively on the training set to avoid data leakage. Finally, the processed dataset was used for predictive modeling.

### 2.5 Feature selection

Feature selection is an essential preprocessing step in model development, helping to reduce overfitting and address the curse of dimensionality in high-dimensional datasets (26). In this study, all feature screening was conducted strictly on the training set to avoid data leakage after splitting the data. For the second-stage regression analysis, the initial feature set included 35 candidate predictor variables.

**Figure 1:**
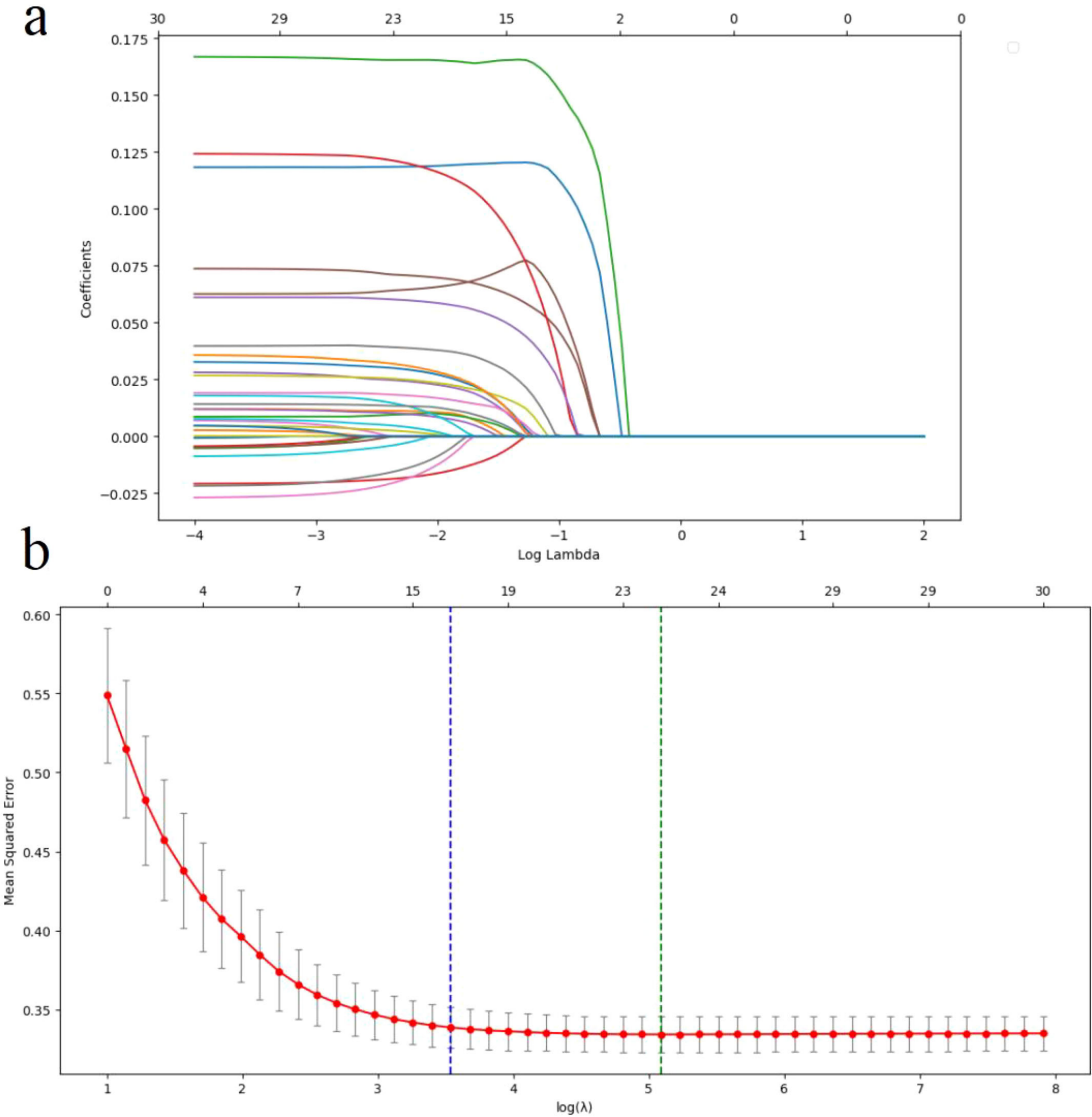
Screening of predictor variables using LASSO regression. **(a)** Coefficient paths of the candidate variables. Each curve illustrates how the coefficient of a variable shrinks as the regularization penalty (log(λ)) increases. **(b)** The 5-fold cross-validation curve for tuning the parameter λ. The left vertical dashed line indicates the optimal log(λ) value that minimizes the mean squared error (MSE), which was used to select the final set of predictive features.

First, univariable screening was applied to the training data using a significance threshold of α = 0.05 as an initial dimensionality-reduction step, leading to the exclusion of three non-significant variables and retaining 32 for further analysis. Next, Least Absolute Shrinkage and Selection Operator (LASSO) regression was employed to identify the most predictive features. As shown in **Figure 1**, the coefficient paths of the candidate variables (**Figure 1a**) demonstrate that, as the regularization penalty (log λ) increases, the coefficients of irrelevant variables gradually shrink toward zero, leaving only the most informative predictors (27). The optimal regularization parameter λ was determined through 5-fold cross-validation (**Figure 1b**), where the value of log λ minimizing the mean squared error (MSE) was selected (28). Ultimately, 23 variables with non-zero coefficients were retained and entered into the subsequent second-stage regression models.

### 2.6 Model development and validation

This study adopted a two-stage modeling approach. In the first stage, school bullying status (yes/no) was used as the dependent variable, and the five social-emotional competency domains were entered as predictors. A LightGBM binary classification model was then developed to examine the relative importance of these domains in distinguishing students with and without bullying experiences. LightGBM was selected because it is well suited for tabular data and can flexibly model complex, potentially non-linear relationships while maintaining strong predictive performance (29). To ensure the robustness of the first-stage model, classification performance was also evaluated on the test set using the area under the receiver operating characteristic curve (AUC), accuracy, and F1-score.

In the second stage, five regression models were constructed: Linear Regression, Decision Tree, K-Nearest Neighbors (KNN), XGBoost, and LightGBM. Hyperparameters for all models were optimized on the training set using grid search with 5-fold cross-validation, and the final configurations are provided in **Supplementary Table 2**. For the XGBoost regression model, additional restrictions were applied to prevent overfitting, including limiting the maximum tree depth, controlling the learning rate, and applying subsampling and feature sampling. The optimal combination of hyperparameters for XGBoost was selected based on cross-validation performance. The best-performing model was selected based on a comparison of evaluation metrics—including R², adjusted R², mean squared error (MSE), and root mean squared error (RMSE)—on the test set. The purpose of this stage was to identify the model that provided the best predictive performance for cooperation atmosphere while allowing subsequent interpretation through SHAP analysis. SHAP was applied to the final selected models to quantify the magnitude and direction of each predictor’s contribution to model output, and mean absolute SHAP values were used to rank predictor importance. These SHAP values were interpreted as indicators of predictive contribution rather than causal effects.

To enhance model robustness and generalizability, the dataset was randomly split into training and test sets at an 8:2 ratio. All continuous predictors were standardized using Z-score normalization. The test set was reserved exclusively for final performance evaluation and was not used during feature selection, preprocessing fitting, or hyperparameter tuning.

### 2.7 Statistical analysis

Continuous variables are presented as mean ± standard deviation. Statistical significance was defined as a two-tailed α level of 0.05. All analyzes were performed using Python 3.12.4 (Python Software Foundation).

## 3 Results

### 3.1 Sample characteristics

The initial sample included 61,010 participants. After removing cases with missing values in key variables, the final analytical sample comprised 7,088 individuals. Among them, 3,355 (47.3%) were female and 3,733 (52.7%) were male. In terms of age, 3,520 participants (49.7%) were 10 years old, and 3,568 (50.3%) were 15 years old.

### 3.2 Comparison of characteristics between victimized and non-victimized students

As shown in **Tables 1** and **Table 2**, students who experienced bullying and those who did not exhibited widespread and significant differences in both social-emotional competencies and a range of psychosocial variables. In terms of social-emotional skills, the non-victimized group scored significantly higher across all 15 skill domains. The disparities were particularly pronounced in areas such as emotional control, stress resistance, and optimism. Furthermore, among the LASSO-selected variables, the victimized group generally demonstrated poorer psychological well-being (e.g., lower life satisfaction and emotional well-being), weaker social support networks (e.g., lower teacher support and sense of school belonging), less healthy behavioral patterns (e.g., more time spent on video games and less participation in social activities), and poorer self-reported health status. These systematic differences clearly outline the multiple risk profiles associated with students who have been bullied.

**Table 1:** Comparison of variables retained after LASSO selection between victimized and non-victimized groups.

Variables	Total (n=7088)	Victimized group (n=3602)	Non-victimized group (n=3486)	*P-value*
Individual behaviors & activities				
Time Spent on Video Games	1.63 ± 0.82	1.71 ± 0.86	1.55 ± 0.77	<0.001
Time Spent Browsing the Internet for Information	1.73 ± 0.65	1.73 ± 0.67	1.74 ± 0.62	0.3797
Time Spent on Physical Exercise Outside of School	2.06 ± 0.78	2.04 ± 0.77	2.08 ± 0.79	0.0398
Participation in Social Activities Outside of School	1.47 ± 0.50	1.42 ± 0.49	1.51 ± 0.50	<0.001
Psychological & emotional states				
Life Satisfaction	7.24 ± 2.27	6.75 ± 2.28	7.75 ± 2.14	<0.001
Total Score of Emotional Well-Being	16.43 ± 4.67	15.57 ± 4.49	17.32 ± 4.68	<0.001
Total Score of Growth Mindset	10.29 ± 2.93	10.02 ± 2.83	10.56 ± 2.99	<0.001
Sense of Security at School	2.61 ± 0.54	2.52 ± 0.56	2.70 ± 0.49	<0.001
Total Score of Sense of Security Outside of School	7.62 ± 1.23	7.40 ± 1.24	7.84 ± 1.18	<0.001
Social relationships & support				
Perceived Friendliness from Classmates	4.14 ± 0.94	3.92 ± 1.01	4.36 ± 0.80	<0.001
Family Intimacy	12.41 ± 2.67	12.04 ± 2.74	12.79 ± 2.55	<0.001
Peer Intimacy	6.97 ± 1.98	6.72 ± 1.97	7.22 ± 1.97	<0.001
Total Score of Parent-Child Conflict	4.97 ± 1.89	5.32 ± 1.94	4.60 ± 1.76	<0.001
Total Score of Perceived Friend Support	12.42 ± 2.76	11.87 ± 2.81	13.00 ± 2.59	<0.001
Teacher Support	10.00 ± 2.33	9.64 ± 2.35	10.36 ± 2.25	<0.001
Total Score of School Belongingness	18.55 ± 3.16	17.73 ± 3.16	19.39 ± 2.93	<0.001
Family & environmental factors				
Number of Books at Home	3.79 ± 1.25	3.76 ± 1.27	3.83 ± 1.23	0.0217
Index of Home Educational Resources	7.57 ± 1.55	7.34 ± 1.65	7.81 ± 1.41	<0.001
Index of Family Material Assets	11.63 ± 3.84	11.55 ± 3.87	11.71 ± 3.80	0.0749
Total Score of Parental Expectation Pressure	7.57 ± 1.80	7.65 ± 1.75	7.49 ± 1.85	<0.001
Highest Parental Education Level (ISCED)	1.93 ± 0.81	1.92 ± 0.82	1.94 ± 0.81	0.3087
Others				
Health Status	2.08 ± 0.96	2.22 ± 0.98	1.94 ± 0.91	<0.001
Competitive Atmosphere	4.62 ± 1.67	4.69 ± 1.59	4.55 ± 1.75	<0.001

**Table 2:** Comparison of social-emotional skills between victimized and non-victimized students.

Variables	Total (n =7088)	Victimized group (n =3602)	Non-victimized group (n =3486)
Assertiveness	25.66 ± 6.03	25.40 ± 6.07	25.93 ± 5.97
Cooperation	33.18 ± 4.48	32.32 ± 4.49	34.07 ± 4.30
Creativity	30.65 ± 5.19	30.24 ± 5.23	31.09 ± 5.12
Curiosity	31.44 ± 4.87	30.92 ± 4.82	31.97 ± 4.86
Emotional control	28.30 ± 6.01	26.96 ± 6.00	29.68 ± 5.69
Empathy	31.47 ± 4.55	30.84 ± 4.50	32.13 ± 4.51
Optimism	30.44 ± 5.97	29.32 ± 6.08	31.60 ± 5.62
Perseverance	30.98 ± 5.33	30.12 ± 5.30	31.87 ± 5.20
Self - control	28.47 ± 4.55	27.73 ± 4.61	29.24 ± 4.36
Sociability	31.06 ± 5.27	30.41 ± 5.36	31.73 ± 5.10
Stress resistance	25.45 ± 7.04	23.93 ± 6.88	27.02 ± 6.86
Tolerance	32.03 ± 4.78	31.55 ± 4.84	32.53 ± 4.67
Trust	30.89 ± 6.11	29.78 ± 6.34	32.04 ± 5.63
Energy	28.07 ± 5.70	27.16 ± 5.70	29.00 ± 5.55
Responsibility	30.67 ± 4.96	29.73 ± 4.94	31.63 ± 4.79

All comparisons between the victimized and non-victimized groups were statistically significant (p < 0.001).

### 3.3 Characteristics of school bullying

**Figure 2:**
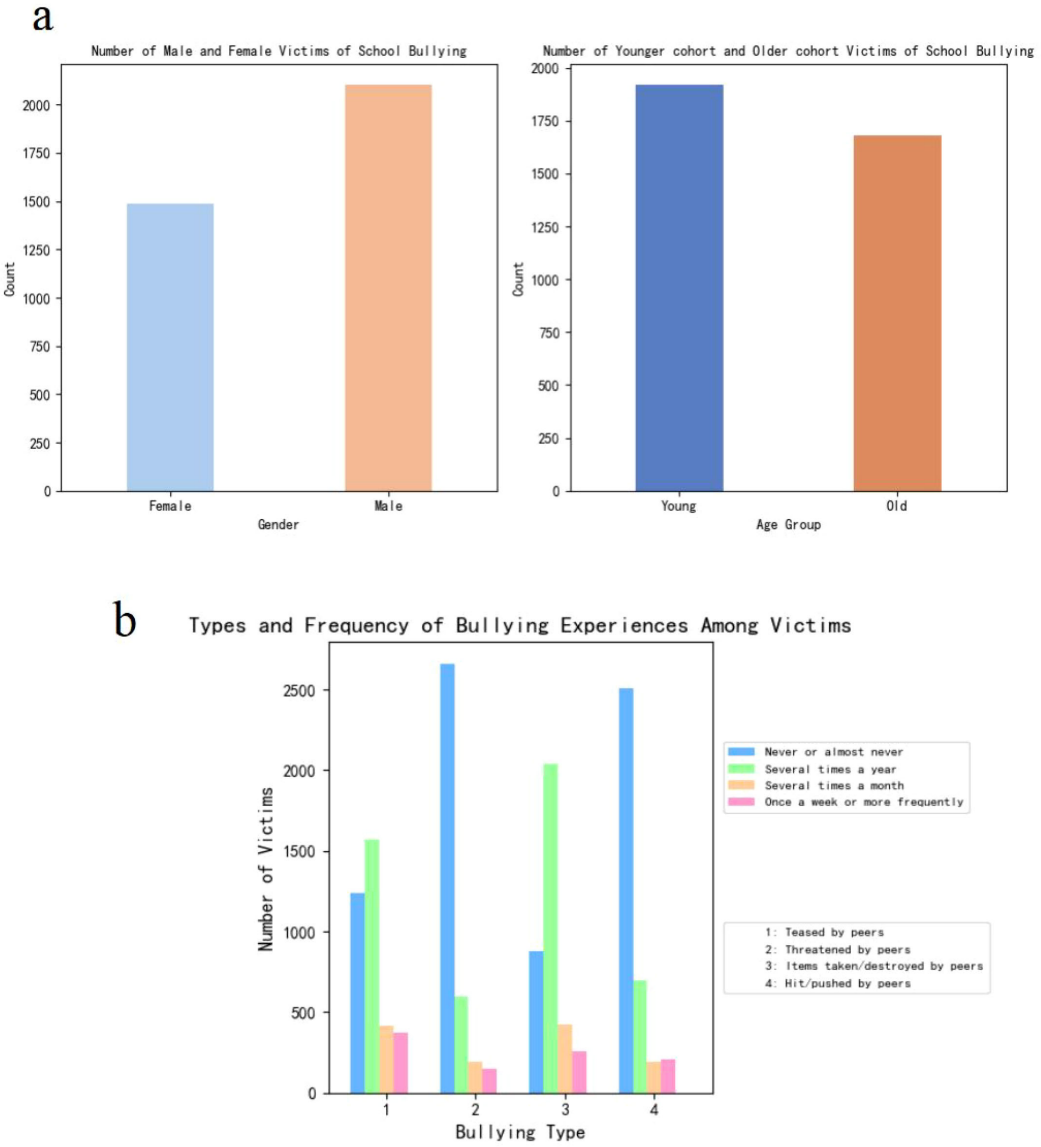
Distribution of school bullying victims by demographic characteristics and bullying type frequency. **(a)** Bar charts showing the number of bullying victims stratified by gender (left) and age cohort (right). **(b)** Stacked bar chart displaying the frequency of different types of bullying experiences among victims.

A total of 3,602 participants reported experiencing school bullying, corresponding to a prevalence rate of 50.8%, while 3,486 (49.2%) reported no bullying experiences. Among the victims, 1,489 (41.3%) were female and 2,103 (58.4%) were male. In addition, 1,920 victims (53.3%) were 10 years old, and 1,682 (46.7%) were 15 years old. The distribution of victims by gender and age is shown in **Figure 2a**. Notably, males reported a significantly higher proportion of victimization than females, and the younger group had slightly more victims than the older group.

**Figure 2b** presents the types and frequency of bullying behaviors. “Being teased by peers” was the most common type and was reported more frequently. Other types, including “being threatened,” “having belongings taken or damaged,” and “being hit or pushed,” were less frequently reported. Most bullying incidents occurred “a few times a year” to “a few times a month,” while experiencing bullying multiple times per week was relatively rare.

### 3.4 Model performance evaluation

This study first examined the interrelationships among all social-emotional skills using Spearman’s rank correlation analysis. As shown in **Supplementary Figure 1**, all skills were positively correlated, with no evidence of high multicollinearity (all |r_s_| < 0.8), supporting their inclusion as distinct yet related predictors in subsequent modeling.

We then comprehensively evaluated model performance at each stage of the two-stage modeling pipeline. The first stage focused on assessing the association between school bullying and social-emotional abilities, rather than building a high-accuracy classifier. Nevertheless, the performance of the first-stage LightGBM classification model was evaluated to ensure the robustness of the variable-importance ranking. On the test set, the model achieved an AUC of 0.666, an accuracy of 0.613, and an F1-score of 0.628, indicating modest but acceptable discriminatory performance for the purpose of variable screening see **Supplementary Tables S3** and **S4** for specific parameters. The LightGBM binary classification model indicated that Cooperation Ability was the social-emotional dimension most strongly associated with bullying status. These results provided key evidence for the subsequent regression modeling in the second phase.

Based on the finding that Cooperation Ability was most strongly associated with bullying status, the closely related variable “Cooperation Atmosphere” was selected as the prediction target in the second phase. Five regression models were constructed: K-Nearest Neighbors (KNN), XGBoost, LightGBM, decision tree, and linear regression. This approach sought to balance robustness and interpretability. Model performance was assessed on both training and testing sets using Mean Squared Error (MSE), Root Mean Squared Error (RMSE), R², and adjusted R² (see **Table 3**).

**Table 3:** Performance evaluation of the regression models on training and testing datasets.

Model	Training set				Test set			
	MSE	RMSE	R²	Adj R²	MSE	RMSE	R²	Adj R²
Linear Regression	0.3305	0.5749	0.3983	0.3959	0.3323	0.5764	0.4186	0.4090
Regression Tree	0.3289	0.5735	0.4014	0.3989	0.3572	0.5977	0.3551	0.3360
k-NN	0.2960	0.5441	0.4612	0.4590	0.3536	0.5964	0.3813	0.3711
XGBoost	0.2724	0.5219	0.5042	0.5022	0.3231	0.5684	0.4347	0.4253
LightGBM	0.2928	0.5411	0.4670	0.4648	0.3239	0.5691	0.4333	0.4239

On the test set, XGBoost performed best (MSE = 0.3231, RMSE = 0.5684, R² = 0.4347, adjusted R² = 0.4253), followed closely by LightGBM (MSE = 0.3239, RMSE = 0.5691, R² = 0.4333, adjusted R² = 0.4239). In contrast, linear regression, decision tree, and k-NN models showed significantly weaker performance. Similar trends were observed on the training set, with XGBoost achieving the highest performance (MSE = 0.2724, RMSE = 0.5219, R² = 0.5042, adjusted R² = 0.5022), followed by k-NN and LightGBM. Linear regression and decision trees again performed poorly.

The two-stage modeling strategy successfully accomplished the research objectives. The first stage identified Cooperation Ability as the variable most strongly associated with bullying. In the second stage, the XGBoost regression model demonstrated robust predictive performance and was selected as the final model for this study.

### 3.5 Feature importance and variable interpretation

**Figure 3:**
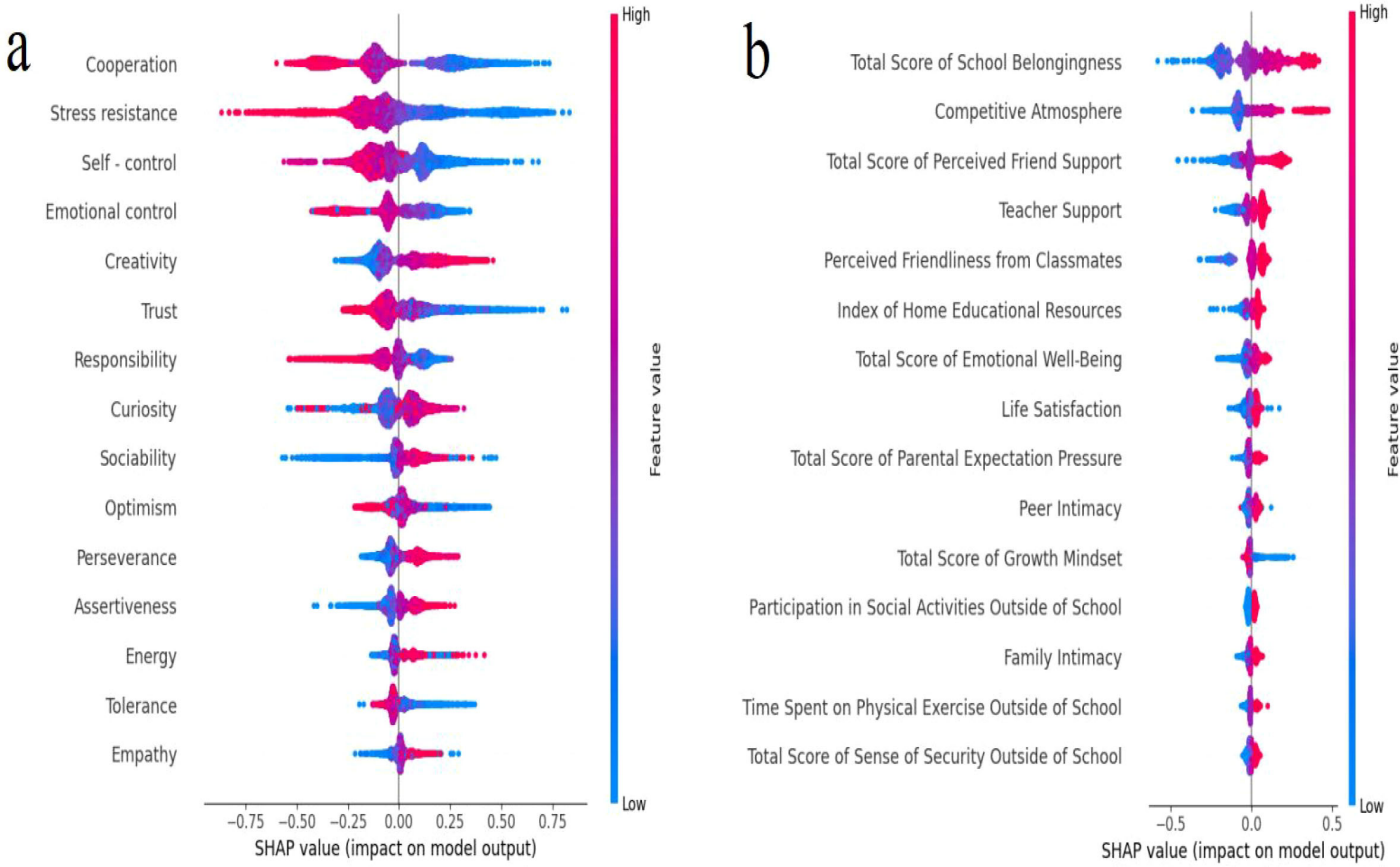
Interpretable machine learning analysis of key predictors using SHAP. **(a)** Summary plot of SHAP values from the first-phase LightGBM binary classification model for predicting school bullying. **(b)** Summary plot of SHAP values from the second-phase model for predicting the Cooperation Atmosphere.

SHAP analysis based on the first-phase LightGBM binary classification model (**Figure 3a**) showed that Cooperation Ability had the highest predictive importance among all socio-emotional variables in distinguishing bullying status. It showed the widest distribution of SHAP values, suggesting the strongest contribution to model predictions. Other significant traits included emotional control, self-discipline, and stress resistance. Furthermore, a strong negative correlation was observed, indicating that higher socio-emotional capability is associated with reduced likelihood of being bullied.

Based on the identified critical role of Cooperation Ability, the second-phase SHAP analysis further explored factors influencing the Cooperation Atmosphere (**Figure 3b**). The results show that school belongingness is the most important predictor, followed by competitive atmosphere and peer support. This underscores the essential roles of social support and campus environmental factors in promoting a supportive Cooperation Atmosphere.

Although competitive atmosphere shows context-dependent and complex effects, school belongingness, peer support, and teacher support consistently exhibited beneficial impacts. Additionally, life satisfaction, emotional health, and family educational resources consistently demonstrated positive predictive value for the Cooperation Atmosphere.

From an interpretable machine learning perspective, the two-stage SHAP analysis not only suggested a potentially protective association between socio-emotional capabilities—especially Cooperation Ability—and school bullying, but also clarified how school social environments and structural factors may shape the Cooperation Atmosphere. This provides both a theoretical basis and quantitative support for developing targeted educational intervention strategies.

### 3.6 Subgroup analysis

**Figure 4:**
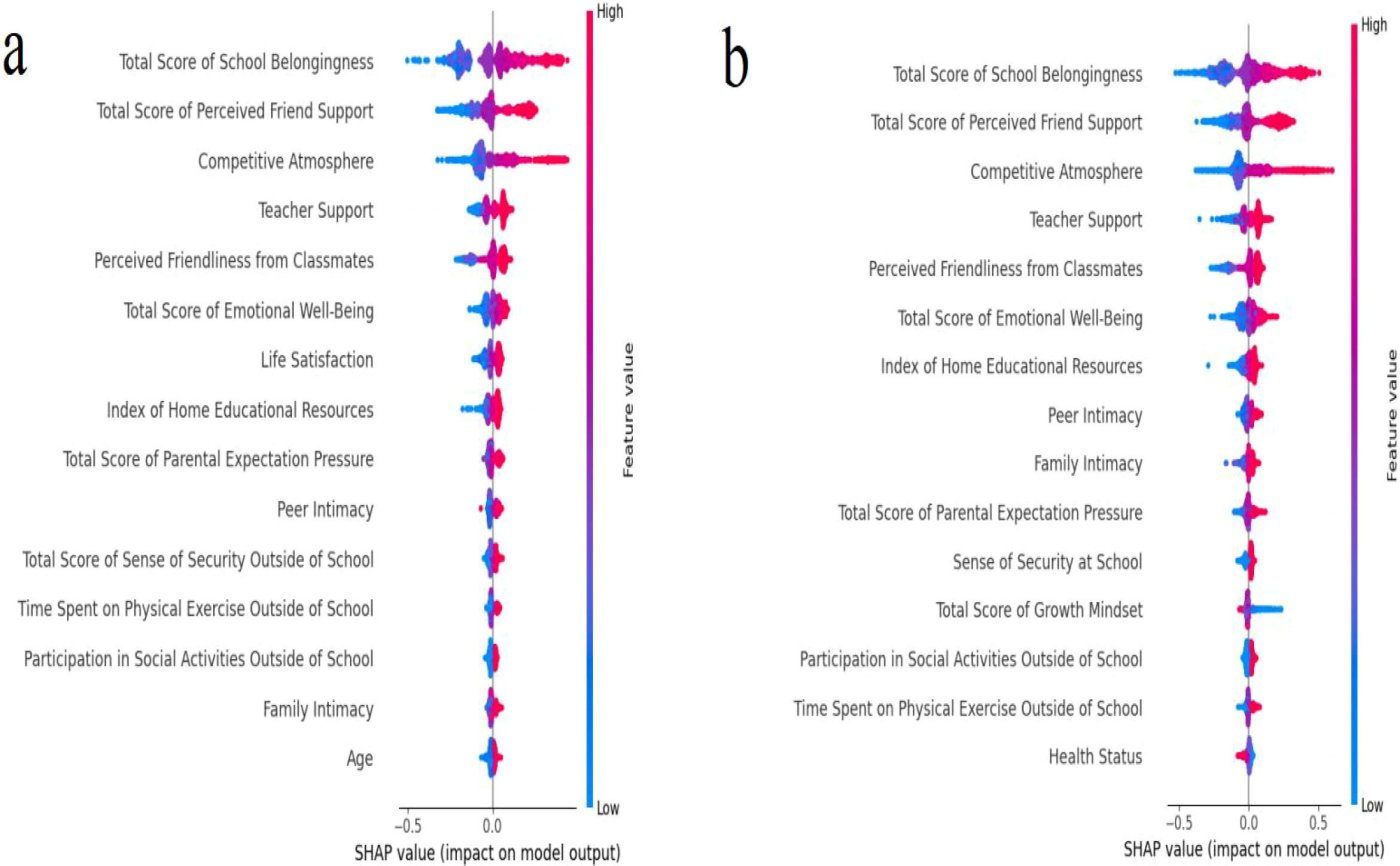
Gender-stratified analysis of predictors for the cooperation atmosphere using SHAP values. **(a)** Key predictors of the Cooperation Atmosphere specific to female students; **(b)** Key predictors of the Cooperation Atmosphere specific to male students.

Subgroup analysis based on SHAP values identified key determinants of the Cooperation Atmosphere and their relative importance across gender categories. As shown in **Figure 4**, factors including teacher support, peer support, and school belongingness were highly ranked for both genders, indicating a consistent influence on the Cooperation Atmosphere among male and female students.

For female students (**Figure 4a**), features with higher SHAP values also included parental expectation pressure, life satisfaction, and sense of safety off-campus. The Cooperation Atmosphere for female students is more influenced by social-emotional support and external expectations, which involve emotional experiences, family expectations, and off-campus safety perceptions.

For male students (**Figure 4b**), the most influential factors were perceived safety on campus, growth mindset, and self-rated health status. The Cooperation Atmosphere among males is more affected by psychological capital and campus environmental factors, reflecting individual psychological attributes, mental and physical health, and perceptions of the school environment.

This gender-stratified SHAP analysis not only confirmed common factors promoting the Cooperation Atmosphere across genders, but also revealed distinct gender-specific predictive patterns. These findings enhance the understanding of how Cooperation Atmosphere is formed and support the design of gender-tailored educational interventions.

## 4 Discussion

The SSES 2019 data indicate that the incidence of school bullying among Chinese adolescents reached 50.8%, significantly exceeding the global average of 32% (3). Among the socio-emotional competencies, Cooperation Ability showed the strongest association with bullying status in the first-stage model. An XGBoost model was developed and demonstrated strong performance in predicting the quality of the Cooperation Atmosphere. SHAP analysis further identified school belongingness, peer support, and competitive atmosphere as significant predictors of the Cooperation Atmosphere, with notable gender-based variations in their influence. These results suggest that bullying may be detrimental to individual Cooperation Ability by disrupting adolescents’ sense of trust, safety, and acceptance in peer interactions, whereas the formation of a positive Cooperation Atmosphere appears to depend on a broader set of relational and school-contextual conditions that support cooperative engagement.

The LightGBM binary classification model indicated that Cooperation Ability was the socio-emotional dimension most strongly associated with school bullying. This pattern may be psychologically meaningful because cooperative behavior depends heavily on interpersonal trust, reciprocity, and willingness to engage in mutually supportive peer interactions, all of which may be particularly vulnerable in bullying contexts. This supports the theoretical framework that bullying—through its inherent violence and exclusion—damages the foundations of peer trust and reciprocity, which are essential for empathy and collaboration (23). Repeated victimization may heighten adolescents’ interpersonal distrust and sense of social threat in peer contexts (21). In turn, disruptions in trustful and reciprocal peer interactions may weaken the expression of cooperative tendencies (23). From a social exchange theory perspective, bullying disrupts the cost–benefit balance in social interactions by introducing psychological and social costs. As a result, adolescents may become less willing to invest in collaborative interactions because peer engagement may be perceived as less rewarding and more risky (30). These findings not only reinforce the established link between bullying and impaired socio-emotional functioning (15, 16) but also highlight the specific relevance of Cooperation Ability in this context, offering further insight into how bullying may be linked to poorer cooperation-related functioning.

Three key factors were identified as predictors of Cooperation Atmosphere: perceived peer support, school belongingness, and competitive atmosphere. School belongingness emerged as the most influential factor. Defined as students’ sense of acceptance, safety, and strong connections within the school community (31), school belongingness satisfies fundamental psychological needs and promotes engagement in collaborative behaviors, as explained by self-determination theory (32). Psychologically, a strong sense of school belonging may reduce perceived interpersonal threat and strengthen students’ expectations of acceptance within the school community, thereby making cooperative peer engagement more likely (7, 31). Furthermore, a strong negative correlation exists between bullying and school belongingness (33). Enhancing belongingness not only reduces the risk of bullying but also buffers its negative psychological effects, such as depression (34). Therefore, fostering school belongingness is both a foundation for building a positive Cooperation Atmosphere and a strategic target for anti-bullying efforts.

The influence of competitive atmosphere on cooperation appears complex and context-dependent. In many studies, excessive competition is considered harmful because it may erode trust and encourage bullying (35), particularly in individualistic cultures that emphasize social comparison (3, 36). However, our study—conducted in a Chinese context—showed a different pattern: competitive atmosphere was positively related to the Cooperation Atmosphere. This finding is consistent with recent evidence from China showing that competitive school climate may be associated with stronger anti-bullying attitudes among Chinese students (35). A possible explanation is that, in Chinese schools, competition may be shaped by cultural values that harmonize competition and cooperation. Despite academic rivalry, collectivist values and institutional guidance may channel competitive impulses into behaviors that benefit the group (37–39).

This finding has important theoretical implications. It suggests that competitive atmosphere should not be understood simply as harmful to peer relationships. Instead, the effect of competition may depend on how it is organized and interpreted within a given cultural and school context. In contrast to much of the Western literature, our findings suggest that competition may coexist with cooperation in Chinese schools. This extends existing theory by showing that the relationship between competition and cooperation is culturally contingent, rather than universally negative.

Peer support also plays a critical role in adolescents’ social development. Peer support may operate through both emotional and behavioral pathways. Emotionally, supportive peer relationships may provide a sense of safety, belonging, and psychological support (40, 41). Behaviorally, supportive peer relationships provide repeated opportunities for reciprocal interaction and prosocial learning (42). Such resilience, in turn, reduces the likelihood of bullying (43, 44). Peer groups also establish social norms that discourage bullying through social learning (45). Healthy peer interactions mitigate egocentrism and advance social-cognitive abilities (42), whereas negative interactions increase bullying risk (46, 47). Thus, peer support is both a protective factor against bullying and a catalyst for a positive Cooperation Atmosphere (48).

Finally, machine learning and SHAP analysis revealed that although core predictors of Cooperation Atmosphere—such as school belongingness and peer support—are consistent across genders, their relative importance and predictive patterns vary. Female students appeared to show greater sensitivity to emotional well-being and external factors (e.g., life satisfaction and off-campus safety), whereas male students appeared to be more strongly associated with personal traits and environmental factors (e.g., growth mindset, self-rated health, and on-campus safety). These differences may reflect gender-related variation in the relative salience of relational-emotional versus competence-environmental cues, although they should be interpreted cautiously because the present findings are based on gender-stratified predictive patterns rather than formal interaction testing. Female students may be more responsive to emotional and interpersonal aspects of the school environment, whereas male students may place greater weight on health, competence-related beliefs, and perceptions of environmental control (49). Consequently, effective interventions may need to account for these gender-specific patterns when seeking to improve the Cooperation Atmosphere and support anti-bullying efforts.

In summary, this study applied a data-driven, two-stage modeling approach to clarify how school bullying is linked to poorer cooperation-related functioning and to identify key social and environmental factors—including gender-specific pathways—that shape the Cooperation Atmosphere. The findings suggest that bullying may disrupt not only observable peer interaction patterns but also the underlying psychological conditions that support cooperation. However, given the cross-sectional design of the SSES 2019 dataset, these associations cannot be interpreted as causal. While predictive models such as XGBoost and SHAP provide insight into important factors related to Cooperation Ability and Cooperation Atmosphere, causal relationships cannot be definitively established. Future longitudinal studies are needed to confirm the direction of these effects and further inform intervention strategies.

Clinically, these findings suggest that therapists working with adolescents who have experienced bullying can focus on improving social-emotional skills, particularly Cooperation Ability. Interventions might include small-group activities to practice collaboration and trust-building exercises, or guided discussions to reinforce positive peer interactions. Strengthening peer support and students’ sense of belonging at school may also help mitigate the negative effects of bullying. Gender-specific patterns indicate that female adolescents may benefit more from interventions addressing emotional well-being and perceived safety, whereas male adolescents may benefit from strategies emphasizing personal coping resources and perceptions of the school environment.

## 5 Conclusions

This study demonstrates that school bullying is closely linked to poorer cooperation-related functioning among Chinese adolescents. Key factors such as school belonging, peer support, and competitive atmosphere play important roles in promoting a positive Cooperation Atmosphere and reducing bullying. Our findings suggest practical actions for educators: implementing social-emotional skill programs, organizing small-group collaboration exercises, strengthening peer support networks, and adapting strategies to gender-specific needs—for example, addressing emotional well-being and perceived safety for girls, and enhancing personal coping skills and perceptions of the school environment for boys. Future research should track changes in the Cooperation Atmosphere over time and assess the impact of tailored interventions. Overall, these results highlight the public health significance of creating cooperative school environments to prevent bullying and support adolescents’ social-emotional development.

## 6 Limitations

This study has several limitations. First, its cross-sectional design does not allow causal inferences. Second, all variables were based on self-reported data, which may be affected by reporting bias. Third, although SHAP analysis improved model interpretability, the identified associations should be understood as predictive rather than causal. Finally, the sample was drawn from adolescents in Suzhou, China, which may limit the generalizability of the findings to other populations or contexts.

## Data Availability

Publicly available datasets were analyzed in this study. This data can be found here: https://www.oecd.org/education/ceri/social-emotional-skills-study/.
