# Supplementary material for: Explainable machine learning unveils the key role of cooperation ability in school bullying and its gender-differentiated impact on cooperative atmosphere
Source: Front Psychiatry. 2026 Jun 4;17:1784847. doi: 10.3389/fpsyt.2026.1784847 (PMC13275709; doi:10.3389/fpsyt.2026.1784847)

**Supplementary Information**

**TableS1.Structure and measurement of the target variables (social-emotional skills)**

**TableS2.Optimal hyperparameters selected for the final predictive models**

****Table S3.** Performance of the first-stage LightGBM classification model**

****Table S4.** Optimal hyperparameters of the first-stage LightGBM classification model**

**Figure S1.**Spearman's rank correlation heatmap of social-emotional skills among the study participants.

**Table S1. Structure and measurement of the target variables (social-emotional skills)**

| ****Construct of Dependent Variable**** | ****Skill Name**** | ****Item Number**** | ****Item Code**** | ****Reverse-Scored Items**** |
| --- | --- | --- | --- | --- |
| **Open-mindedness skills** | **Curiosity** | 04 | CUR | STA_CURO6 |
|  | **Tolerance** | 10 | TOL | STA_TOL06 |
|  | **Creativity** | 16 | CRE | STA_CRE03/07/08 |
| **Task performance** | **Responsibility** | 07 | RES | STA_RES01/03/04/07/08 |
|  | **Self-control** | 17 | SEL | STA_SEL05/08 |
|  | Perseverance | 13 | PER | STA_PER03/05/06 |
| **Engaging with others** | **Sociability** | 02 | SOC | STA_SOC04/08 |
|  | **Assertiveness** | 14 | ASS | STA_ASS05 |
|  | **Energy** | 08 | ENE | STA_ENE03/04/06/07 |
| **Collaboration** | **Empathy** | 05 | EMP | STA_EMP08 |
|  | **Trust** | 11 | TRU | STA_TRU05 |
|  | **Cooperation** | 18 | COO | STA_COO04 |
| **Emotional regulation** | **Stress resistance** | 15 | STR | STA_STR02/03/04/05/06/07/08 |
|  | **Optimism** | 09 | OPT | STA_OPT01/08 |
|  | **Emotional control** | 03 | EMO | STA_EMO03/05/06/08 |

****Table S2**. Optimal hyperparameters selected for the final predictive models**

| ****Regression Tree**** | {'max_depth': 5, 'min_samples_leaf': 10, 'min_samples_split': 2} |
| --- | --- |
| ****k-NN**** | {'n_neighbors': 15, 'p': 1, 'weights': 'distance'} |
| **XGBoost** | {'colsample_bytree': 0.8, 'learning_rate': 0.1, 'max_depth': 3, 'n_estimators': 100, 'subsample': 0.8} |
| **LightGBM** | {'colsample_bytree': 0.8, 'learning_rate': 0.1, 'max_depth': 3, 'n_estimators': 50, 'num_leaves': 31, 'subsample': 0.8} |

****Table S3. Performance of the first-stage LightGBM classification model****

| ****Model**** | ****Dataset**** | ****AUC**** | ****Accuracy**** | ****F1-score**** |
| --- | --- | --- | --- | --- |
| LightGBM (Stage 1) | **Test set** | **0.666** | **0.613** | **0.628** |

****Table S4. Optimal hyperparameters of the first-stage LightGBM classification model****

| LightGBM | **{'colsample_bytree': 0.9, 'learning_rate': 0.05, 'max_depth': 3, 'n_estimators': 200, 'num_leaves': 31, 'subsample': 0.9}** |
| --- | --- |

**Figure S1.**Spearman's rank correlation heatmap of social-emotional skills among the study participants.


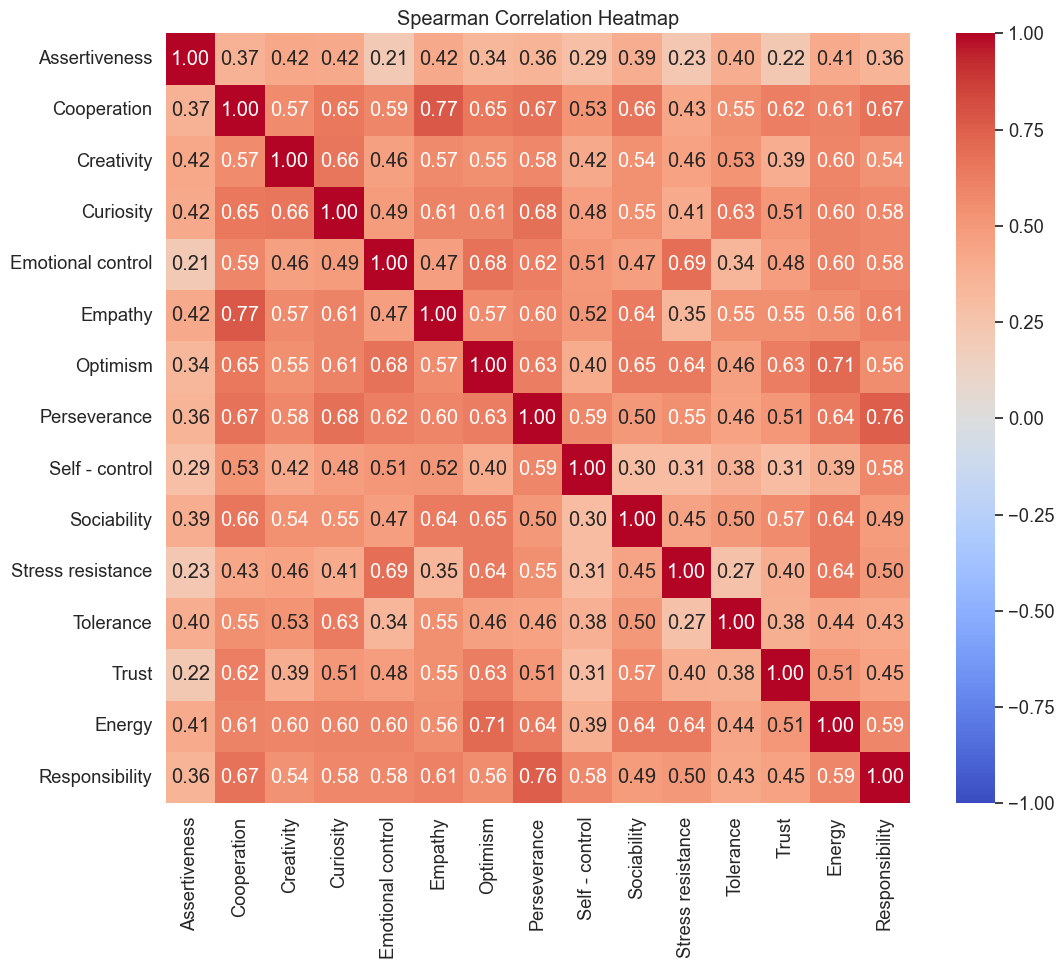

Supplement: Supplementary file 1 [file Supplementaryfile1.doc]
